# Efficient two-step photocarrier generation in bias-controlled InAs/GaAs quantum dot superlattice intermediate-band solar cells

**DOI:** 10.1038/s41598-017-05494-8

**Published:** 2017-07-19

**Authors:** T. Kada, S. Asahi, T. Kaizu, Y. Harada, R. Tamaki, Y. Okada, T. Kita

**Affiliations:** 1 Department of Electrical and Electronic Engineering, Graduate School of Engineering, Kobe University, 1-1 Rokkodai, Nada, Kobe 657-8501 Japan; 2 Research Center for Advanced Science and Technology (RCAST), The University of Tokyo, 4-6-1 Komaba, Meguro-ku, Tokyo 153-8904 Japan

## Abstract

We studied the effects of the internal electric field on two-step photocarrier generation in InAs/GaAs quantum dot superlattice (QDSL) intermediate-band solar cells (IBSCs). The external quantum efficiency of QDSL-IBSCs was measured as a function of the internal electric field intensity, and compared with theoretical calculations accounting for interband and intersubband photoexcitations. The extra photocurrent caused by the two-step photoexcitation was maximal for a reversely biased electric field, while the current generated by the interband photoexcitation increased monotonically with increasing electric field intensity. The internal electric field in solar cells separated photogenerated electrons and holes in the superlattice (SL) miniband that played the role of an intermediate band, and the electron lifetime was extended to the microsecond scale, which improved the intersubband transition strength, therefore increasing the two-step photocurrent. There was a trade-off relation between the carrier separation enhancing the two-step photoexcitation and the electric-field-induced carrier escape from QDSLs. These results validate that long-lifetime electrons are key to maximising the two-step photocarrier generation in QDSL-IBSCs.

## Introduction

Development of solar cells (SCs) is one of the most challenging problems in the field of renewable energy. In particular, low-cost, high-performance SCs have been highly sought for use in practical applications. The energy conversion efficiency under a 1-sun solar irradiance achieved by conventional single-junction SCs has already reached 28.8%^1^ for thin-film GaAs crystals, which is close to the theoretically achievable so-called Shockley–Queisser limit^2^. In conventional single-junction SCs, the energy conversion efficiency depends on the bandgap energy of the corresponding semiconductor material^2, 3^. When photons with energies higher than the bandgap energy are absorbed, the output voltage coincides with the quasi Fermi-level splitting in the band gap, and the photocurrent is proportional to the number of absorbed photons. Here, carriers excited above the band gap lose their excess energy in the process of thermalisation. Conversely, photons with energies below the bandgap energy pass through the SC without being absorbed. To realise higher conversion efficiencies, new techniques are needed for reducing the above-mentioned losses. Intermediate-band SCs (IBSCs) are one of the most promising SC structures^4, 5^.

An IBSC is a *p-i-n* single-junction SC with an intermediate band (IB) in the band gap of the intrinsic layer, which can absorb photons with energies below that of the band gap, causing two additional optical transitions, from the valence band (VB) to the IB and from the IB to the conduction band (CB), in addition to the conventional VB-to-CB transition^5^. This allows IBSCs to generate an additional photocurrent while maintaining the output voltage of single-junction SCs. According to previous theoretical work, the energy conversion efficiency of IBSCs is ~48% under the condition of 1-sun non-concentrated irradiation with AM1.5, while the energy conversion efficiency is ~67% under the condition of maximal concentration^6^. Despite such a high predicted efficiency, several issues hinder realising ideal carrier dynamics in IBSCs. The key issue limiting the efficiency is the IB-to-CB intersubband absorption strength. The conversion efficiency is very sensitive to the intersubband absorption coefficient; the intersubband absorption coefficient of 1,000 /cm attains the efficiency of 40%^4^, and the coefficient of 10,000 /cm is essential for realising SCs with an extremely high efficiency of 50%. The intersubband absorption coefficient *α*_IC_ is given by ${f}_{{\rm{IB}}}{\alpha }_{{\rm{IC}}}^{{\rm{1}}}$, where *f*_IB_ is the state filling factor of the IB and ${\alpha }_{{\rm{IC}}}^{{\rm{1}}}$ is the absorption coefficient when the initial IB state is completely filled^4, 7^. According to the optical selection rule for the intersubband transition, a three-dimensionally confined system such as quantum dots (QDs) is promising for enhancing the absorption strength for light rays normally incident onto a SC surface. Thus, significant amount of research has been performed on QD-SCs. In particular, InAs QDs embedded in GaAs have been used for forming the IB because the quantisation level is appropriate for the IB in GaAs, and solid findings of two-step photon absorption have been reported^7–14^. Recently, techniques for controlling the electron density in the IB have attracted significant attention, because *α*_IC_ is proportional to *f*_IB_^4^. High density of electrons improves *α*_IC_, implying that long-lifetime electrons in the IB are indispensable for realising high conversion efficiency SCs. The conversion efficiency significantly reduces with shortening the electron lifetime^4^. Electron-hole recombination, thermal excitation, and tunnelling escape all limit the electron lifetime. The probability of thermal excitation can be reduced by introducing a high-potential barrier^15, 16^, and tunnelling carrier escape rate can be reduced by controlling the internal electric field^13, 17^. Besides, the internal electric field influences carrier drift velocity and carrier capture in QDs as well as state filling of the IB^8, 9^. Controlling the lifetime of electrons is very challenging. Several approaches based on the concept of ratcheting excited carriers in the IB have been proposed^18^ and demonstrated using InAs/GaAs QD superlattices (QDSLs)^14^ and InAs QDs embedded in the GaAs/Al_0.3_Ga_0.7_As quantum well^15, 16^. Stacked QDs, separated by a thin barrier layer, form a superlattice (SL) structure with energy minibands^12, 19–21^. Thereby, excited electrons and holes can be spatially separated along the SL in the internal electric field of the SC, yielding an extended, long carrier recombination lifetime and thus enhancing the intersubband transition strength^14^. In other words, the internal electric field can change the intersubband transition strength.

In this work, we systematically studied two-step photocarrier generation in InAs/GaAs QDSL-IBSCs. We measured the external quantum efficiency (EQE) and its enhancement caused by an additional infrared (IR) light illumination as a function of the electric field intensity. We compared the experimental results with theoretical calculations taking into account rate equations describing transitions in the QDSL states.

## Results

**Figure 1:**
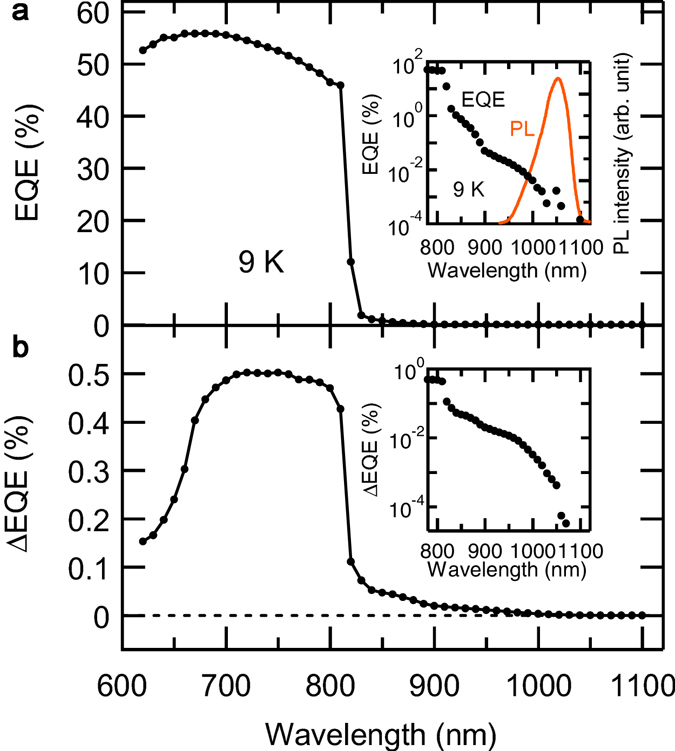
EQE and ΔEQE spectra for QDSL-IBSC. (**a**) EQE spectrum measured at 9 K. (**b**) ΔEQE spectrum measured at 9 K. The inserts show magnified plots in the subbandgap region. PL spectrum was measured for the interband excitation of 800 nm.

Figure 1(a) shows the EQE and photoluminescence (PL) spectra obtained for the short circuit condition. The EQE spectrum exhibits a clear excitonic absorption edge of GaAs at 820 nm (1.52 eV). The inset shows a magnified view of the EQE spectrum in the region below the bandgap energy. The EQE signal extends towards longer wavelengths. A weak edge at ~880 nm is attributed to the interband photoexcitation of the InAs wetting layer. Electrons excited in the shallow wetting layer states can be easily extracted by the internal electric field because the confinement barrier’s height is lowered in the presence of the electric field. The EQE signal further decreases exponentially towards longer wavelengths, up to the QD state^14^. The ground state (GS) PL peak is visible at 1,054 nm (1.18 eV), and the tail structure on the shorter-wavelength side of the PL peak is attributed to the excited state (ES). Here, electrons excited into the QDSL states cannot easily escape owing to the strong confinement. In other words, electrons are efficiently accumulated in the IB at 9 K, which is suitable for studying two-step photocarrier generation in QDSL-IBSCs. Figure 1(b) shows the ΔEQE spectrum measured at 9 K. The ΔEQE signal becomes strong in the 700–800 nm range, where the first interband excitation occurs between the VB and CB of GaAs. Some excited electrons in the CB relax into the QDSL states and are re-pumped by the second IR excitation. This is a typical loss pathway in IBSCs. The rapid reduction in the ΔEQE signal for wavelengths below ~700 nm stems from a shallow penetration depth of the first interband excitation light. This reduction can be alleviated by introducing a window layer atop the GaAs *p-i-n* structure. Below the GaAs band gap, the ΔEQE signal is clear. This occurs owing to the two-step photoexcitation of subbandgap states of the wetting layer and QDSLs. Here, it is of note that the second subbandgap absorption efficiently occurs when electrons are pumped into the ES of QDSLs in the first excitation step. Previously^14^, we reported a similar phenomenon and attributed it to the separation of carriers in the ES miniband owing to the internal electric field. The electron lifetime was extended by inhibiting electron-hole recombination, enhancing the second subbandgap absorption.

**Figure 2:**
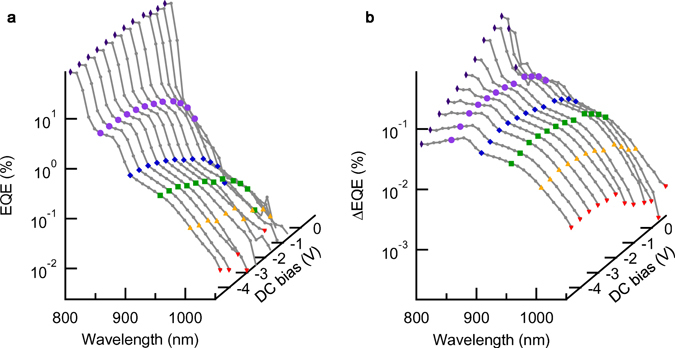
EQE and ΔEQE spectra measured at different applied DC bias voltages. (**a**) EQE spectra in the subbandgap region measured by applying the external DC bias voltage. The solid symbols indicate the EQE at 800, 850, 900, 950, 1,000, and 1,050 nm of the interband excitation, respectively. (**b**) ΔEQE spectra in the subbandgap region measured with the additional subbandgap excitation of 0.5 eV. The DC bias was changed from 0.8 V to −4.5 V.

**Figure 3:**
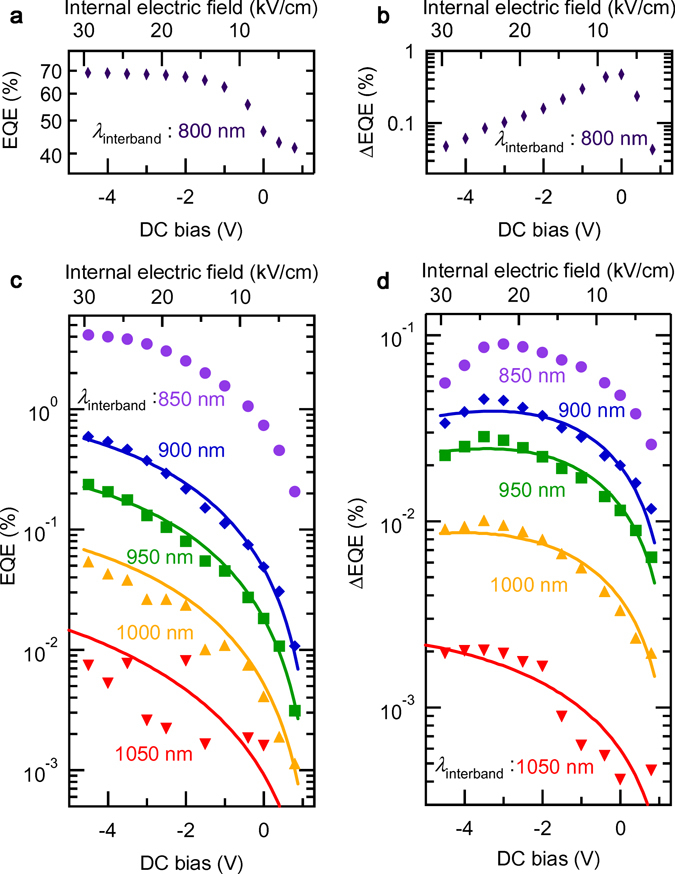
DC-bias dependence of the EQE and ΔEQE signal intensities detected at several wavelengths of the first interband excitation. (**a**) EQE and (**b**) ΔEQE at 800 nm as a function of the internal electric field. (**c**) EQE and (**d**) ΔEQE represent the results at 850, 900, 950, 1,000, and 1,050 nm. These data are taken at 9 K.

The EQE and ΔEQE spectra measured at different applied DC bias voltages are shown in Fig. 2(a) and (b). Figure 3(a),(b),(c) and (d) summarise the DC-bias dependence of the EQE and ΔEQE signal intensities detected at several wavelengths of the first interband excitation. Solid symbols indicate the experimental data, while solid lines are the calculated results that are discussed later. With increasing the applied DC bias voltage, the internal electric field increases, improving the carrier collection efficiency^22^ by suppressing the carrier recombination. Thereby, the EQE signal intensity monotonically increases with increasing the electric field. In particular, the EQE at 800 nm above the GaAs band gap reaches 69% at −4.5 V (30 kV/cm) (see Fig. 3(a)). A similar trend was observed for the EQE measured at subbandgap wavelengths (Fig. 3(c)). The EQE signal intensities at subbandgap wavelengths are much weaker than that at 800 nm, owing to the small absorption coefficient and carrier confinement in QDSLs. Conversely, ΔEQE reveals interesting features. The ΔEQE signal above the GaAs band gap is the strongest in the zero-bias condition (see Fig. 3(b)). With increasing the forward bias, ΔEQE rapidly decreases because the carrier collection efficiency decreases and the carrier recombination in QDSLs becomes predominant. On the other hand, with increasing the reverse bias, a strong electric field prevents excited electrons from relaxing into the QDSL states, therefore decreasing the ΔEQE. In contrast to this trend for ΔEQE excited above the GaAs band gap, the ΔEQE signal intensity for the subbandgap interband excitation for wavelengths longer than 850 nm gradually increases with increasing the internal electric field and saturates as shown in Fig. 3(d). Moreover, the signal starts to decrease after reaching a maximum. That the ΔEQE signal intensity increases with increasing the electric field intensity is strange, because an increase in the EQE signal with increasing the electric field intensity implies a reduction in the electron density in the IB, which should reduce the second subbandgap absorption^23, 24^. This counterintuitive observation can be interpreted by considering the separation of carriers in the QDSL miniband. With increasing the electric field, photoexcited electrons and holes are separated in the ES miniband, and the electron lifetime is extended by inhibiting the recombination of electrons with holes, enhancing the second subbandgap absorption. This causes the increase in the ΔEQE signal intensity with increasing the electric field. However, when the electric field becomes too strong, extraction of electrons from the IB becomes dominant, reducing the second subbandgap absorption.

**Figure 4:**
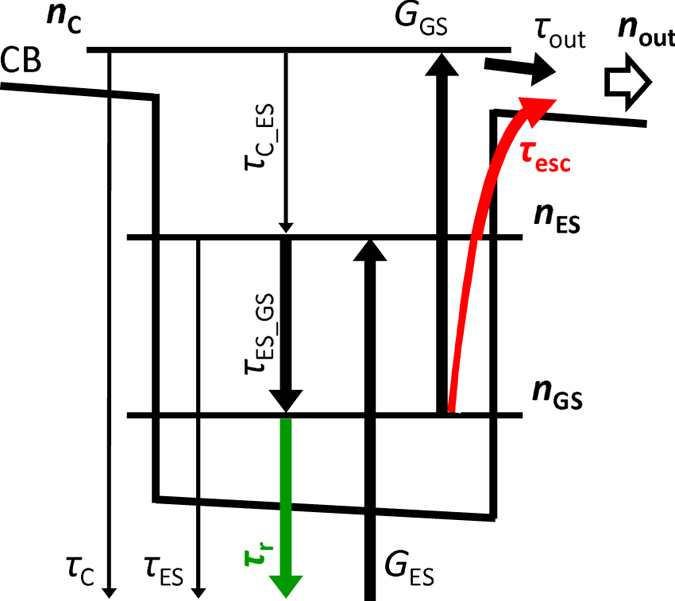
Schematic diagram of the simulation model used in this work. GS and ES of QDSLs were taken into account in the calculations. Extracted current density *n*_out_ was calculated as a function of the internal electric field and the interband excitation wavelength.

To clarify the effects of the internal electric field on EQE and ΔEQE, we performed theoretical calculations of carrier-extraction efficiency (CEE) for carriers generated by the interband photoexcitation at a certain wavelength. CEE based on the model of dynamics of photoexcited carriers in a QD. We calculated the CEE of photoexcited carriers as a function of the electric field intensity. The model used here is illustrated in Fig. 4. We accounted for the interband excitation in the ES of a QD, the intersubband excitation in the QD, the energy relaxation in the QD, thermal and/or electric field induced electron escape from the QD states, and carrier recombination. We considered two quantised levels in the QD and one extended state above the GaAs bandgap. Here, we included the carrier separation effect in the recombination lifetime as a fitting parameter.

Rate equations for the electron densities per unit area, *n*_GS_, *n*_ES_, and *n*_C_ in the GS, ES, and CB states of the QDSL, are given by

1 $$\frac{\partial {n}_{C}}{\partial t}={G}_{GS}-\frac{{n}_{C}}{{\tau }_{C\_\mathrm{ES}}}-\frac{{n}_{C}}{{\tau }_{{\rm{out}}}}-\frac{{n}_{C}}{{\tau }_{{\rm{C}}}}$$

2 $$\frac{\partial {n}_{{\rm{ES}}}}{\partial t}={G}_{{\rm{ES}}}-\frac{{n}_{{\rm{ES}}}}{{\tau }_{\mathrm{ES}\_\mathrm{GS}}}(1-\frac{{n}_{{\rm{GS}}}}{DO{S}_{GS}})+\frac{{n}_{C}}{{\tau }_{C\_\mathrm{ES}}}-\frac{{n}_{{\rm{ES}}}}{{\tau }_{{\rm{ES}}}}-\frac{{n}_{{\rm{ES}}}}{{\tau }_{{\rm{esc}}}}$$

3 $$\frac{\partial {n}_{{\rm{GS}}}}{\partial t}=-{G}_{{\rm{GS}}}+\frac{{n}_{{\rm{ES}}}}{{\tau }_{\mathrm{ES}\_\mathrm{GS}}}(1-\frac{{n}_{{\rm{GS}}}}{DO{S}_{{\rm{GS}}}})-\frac{{n}_{{\rm{GS}}}}{{\tau }_{{\rm{r}}}}-\frac{{n}_{{\rm{GS}}}}{{\tau }_{{\rm{esc}}}}$$

*DOS*_GS_ and *DOS*_ES_ are the densities of states for the GS and ES, respectively. *DOS*_GS_ and *DOS*_ES_ were both 1.8 × 10^11^/cm^2^ according to the in-plane QD density of 1 × 10^10^/cm^2^ ^12^, the layer number of 9, and the spin degeneracy. Here, we simply assumed that the ES is a non-degenerated state owing to the non-cubic shape of the InAs/GaAs QDs. *τ*_C_ES_ and *τ*_ES_GS_ are the energy relaxation lifetimes from the CB to the ES and from the ES to the GS, respectively. *τ*_ES_ and *τ*_C_ are the recombination lifetimes in the ES and CB, respectively. *τ*_*r*_ is the time constant of the interband recombination in the GS. *τ*_esc_ is the time constant of thermal and/or electric field induced electron escape. *τ*_out_ is the time constant of the electron transfer from the extended state of the QDSL to GaAs. Here, the second terms on the right hand side of Eq. (2) and Eq. (3) represent the energy relaxation rate from the ES to the GS, respectively. The Pauli blocking effects of the GS are taken into account, while we ignored the Pauli blocking effects of the ES because the calculated electron density was approximately nine order smaller than the *DOS*_ES_. *G*_ES_ and *G*_GS_ are the interband and intersubband photocarrier generation rates in the QD, respectively. They are given by

4 $${G}_{ES}={P}_{{\rm{ES}}}\{1-\exp (-{\alpha }_{{\rm{ES}}}{t}_{{\rm{QDSL}}})\}$$

and

5 $${G}_{{\rm{GS}}}={P}_{{\rm{GS}}}\{1-\exp (-\frac{{n}_{{\rm{GS}}}}{DO{S}_{{\rm{GS}}}}{\alpha }_{{\rm{GS}}}{t}_{{\rm{QDSL}}})\}$$

*P*_ES_ and *P*_GS_ are the incident photon fluxes of the interband and intersubband excitations, respectively. They were calculated from the excitation power density by considering the reflectivity at the SC surface and the photon energy. The reflectivity was 31% for the interband excitation and 29% for the intersubband excitation^25^. The interband excitation power was changed from 153 to 175 μW/cm^2^. The intersubband excitation power was fixed at 520 mW/cm^2^. *α*_ES_ and *α*_GS_ are the absorption coefficients of the ES and the GS, respectively. *t*_QDSL_ is the QDSL thickness, set to 38 nm.

Equations (1), (2), and (3) should evaluate to zero in the steady state. We obtained each carrier density by substituting Eqns (4) and (5) into Eqns (1)–(3). Finally, the total number of the collected electrons is given by

6 $${n}_{{\rm{out}}}=\frac{{n}_{{\rm{C}}}}{{\tau }_{{\rm{out}}}}+\frac{{n}_{{\rm{ES}}}}{{\tau }_{{\rm{esc}}}}+\frac{{n}_{{\rm{GS}}}}{{\tau }_{{\rm{esc}}}}$$

We calculated the EQE from the photocurrent density given by *e* × *n*_out_ divided by the incident photon density *P*_ES_, where *e* is the electron charge. ΔEQE corresponds to a change in the EQE when accounting for the intersubband excitation. We assumed that *τ*_C−ES_, *τ*_ES_GS_, *τ*_C_, *τ*_ES_, and *α*_GS_ are constant. We used the values of 0.1 μs for *τ*_C_ES_, *τ*_C_, and *τ*_ES_, and 0.1 ns for *τ*_ES_GS_^26, 27^. The values of ΔEQE were calculated as functions of *τ*_r_, *τ*_esc_, and *α*_ES_. Here, *α*_ES_ varied with the interband excitation wavelength, as shown in Table 1. It is noted that *τ*_out_, *τ*_r_, and *τ*_esc_ vary with the electric field *F*. *τ*_r_ obeys an empirical relationship given by ${\tau }_{{\rm{r}}}={\tau }_{{\rm{0}}}(1+{C}_{{\rm{r}}}\times F)$, where *C*_r_ is a constant depending on the interband excitation wavelength^14^. Previously, we estimated *τ*_0_ as 1.3 ns. *τ*_esc_ and *τ*_out_ were assumed to be proportional to 1/*F*.

**Table 1:** Evaluated absorption coefficients in the QDSL.

Interband excitation wavelength (nm)	900	950	1,000	1,050
*α*_ES_ (/cm)	8,000	3,300	1,150	670
*α*_GS_ (/cm)	650			

**Figure 5:**
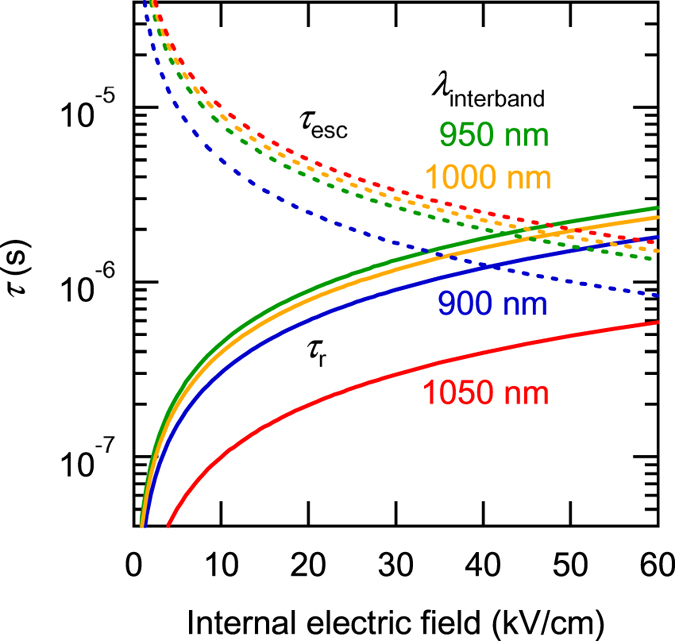
Internal electric field dependence of the evaluated time constants of *τ*_r_ and *τ*_esc_ at 900, 950, 1,000, and 1,050 nm as a function of the internal electric field.

**Figure 6:**
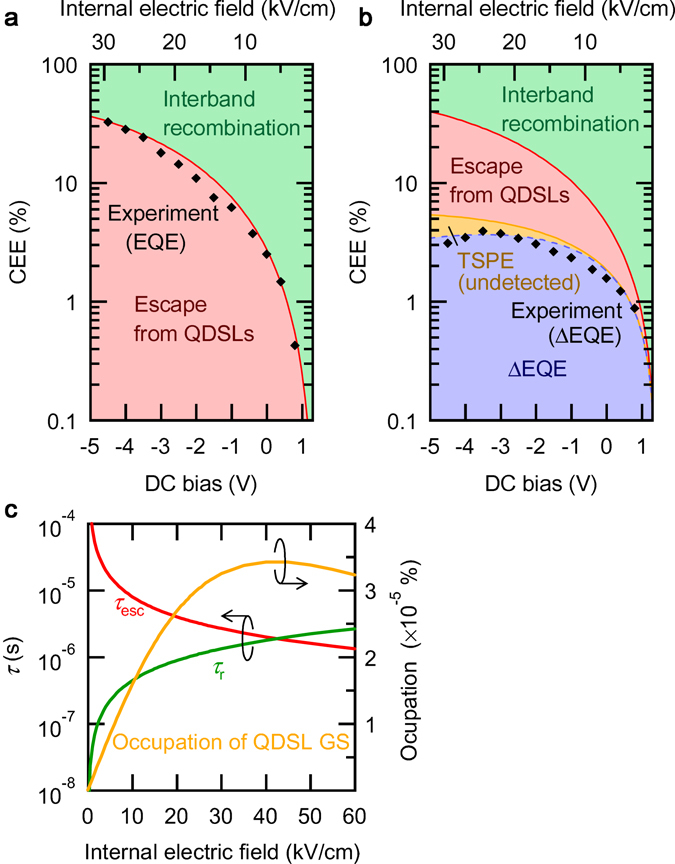
Calculated carrier-extraction efficiency for carriers generated by the interband photoexcitation at 950 nm. (**a**) and (**b**) show the results obtained without and with the intersubband excitation, respectively. The solid symbols indicate the experimental results normalised by the interband photoexcited carrier density at 950 nm. (**c**) summarises the evaluated time constants a function of the internal electric field.

Figure 3 compares the DC bias dependence of the measured EQE and ΔEQE with the calculated results. The calculated results, indicated by the solid lines, reproduce well the measured data. Here, we used the fitting parameters listed in Table 1, and the evaluated variations of *τ*_r_ and *τ*_esc_ are shown by the solid and dashed lines in Fig. 5. *τ*_r_ was calculated by using the empirical relation ${\tau }_{{\rm{r}}}={\tau }_{{\rm{0}}}(1+{C}_{{\rm{r}}}\times F)$. *τ*_r_ monotonically increased from 1.3 ns with increasing the electric field. With increasing the electric field, *τ*_r_ reached the order of microseconds, owing to the carrier separation in the ES miniband, which became maximal at 950 nm, corresponding to the ES miniband excitation. On the other hand, *τ*_esc_, capturing the time constant of the carrier escape from the QDSL, decreased with decreasing the interband excitation wavelength, as the final energy level of the excitation approached the potential barrier’s height. Figure 6 compares typical EQE and ΔEQE measured at 950 nm (corresponding to the ES excitation), with the results of detailed calculations of CEE. The black diamonds in Fig. 6 indicate the experimental results normalised by the number of generated electrons. Figure 6(a) shows the calculated CEE when conducting the only interband excitation at 950 nm. The measurable photocurrent corresponds to the red area attributed to the field-induced carrier escape from the QDSL. The carrier escape monotonically increases with increasing the electric field. The remaining CEE indicated by green is owing to the recombination loss in the QDSL. A stronger electric field more easily extracts carriers and more significantly reduces the recombination loss. The experimental results are in an excellent agreement with the calculated results. When IR illumination is added, the intersubband transition occurs and the photocurrent breaks down into four regions, as shown in Fig. 6(b). The appearance of the blue and yellow areas is attributed to the additional IR illumination. The blue area corresponds to a measurable ΔEQE. Note that ΔEQE increases as much as the interband recombination loss decreases. On the other hand, the yellow area represents the contribution of the additional IR illumination for electrons originally escaping from the QDSL in the electric field, which cannot be detected in the experiment synchronised with the modulated IR illumination. Thus, the experimental data indicated by the diamonds coincide with the curve given by the blue area. ΔEQE increases with increasing the electric field, because the field improves the separation of carriers in the QDSL miniband and extends their lifetime, despite decreasing the electron density in the QDSL states, as shown in Fig. 6(a). ΔEQE is maximal and decreases with further increasing the electric field above ~25 kV/cm. This suggests that a remarkable extraction of carriers in such a strong electric field suppresses any additional intersubband excitation. These results indicate that there is a trade-off between the lifetime extension and the field-induced carrier extraction. Thus, there is an optimal electric field maximising the two-step photocarrier generation depending on the electron population density in the GS.

Figure 6(c) compares the internal electric field dependence of the evaluated time constants *τ*_r_ and *τ*_esc_ and the carrier occupation in the QDSL GS. *τ*_r_ reaches ~1.1 μs at 25 kV/cm, exhibiting the maximal ΔEQE. Long-lifetime electrons can be attained by inhibiting their recombination with holes. On the other hand, *τ*_esc_, representing the time constant for carrier escape from the QDSL, decreases as the electric field increases. The values of these two time constants become the same at ~40 kV/cm, where the carrier occupation and, therefore, the two-step photocurrent generation becomes maximal. It is noted that ΔEQE varies with this carrier occupation in the GS. Furthermore, as shown in Fig. 3, the electric field corresponding to the maximal ΔEQE slightly shifts with the interband excitation wavelength. The ΔEQE maximum shifts towards stronger electric fields for longer excitation wavelengths. This feature was also reproduced in Fig. 5 as a change in the carrier occupation depending on the electron population density in the GS.

## Discussion

We systematically studied two-step photocurrent generation in InAs/GaAs QDSL-IBSCs as a function of the electric field intensity. We found that ΔEQE caused by the two-step photoexcitation is maximal at a reversely biased electric field, while EQE owing to the interband photoexcitation increases monotonically with increasing the electric field intensity. With increasing the electric field intensity, photoexcited electrons and holes are separated in the ES miniband, and the electron lifetime is extended by inhibiting their recombination with holes, thus increasing the ΔEQE with increasing the electric field intensity. However, when the electric field intensity becomes too strong, electron extraction from the IB becomes dominant and reduces the second subbandgap absorption. The carrier occupation in the GS is balanced by the electron lifetime extension and the field-induced carrier extraction. The maximal occupation in the GS yields the optimal condition for IBSCs. These results clarify that long-lifetime electrons owing to the field-induced carrier separation are key for maximising the conversion efficiency of IBSCs.

As mentioned for Fig. 3(d), the ΔEQE signal intensity for the subbandgap interband excitation for wavelengths longer than 850 nm shows the different position of the peak ΔEQE. ΔEQE gradually increases with increasing the internal electric field and shows the maximum at which the carrier occupation of QDSL GS becomes maximal when the electron lifetime is equal to the time constant for carrier escape from the QDSL. In order to place the peak at the optimal point for generating power, the time constant of the interband recombination in the GS is required to be extended furthermore. In our QDSL-IBSC, photoexcited electrons and holes are separated in the ES miniband of QDSL. It is noted that the GS of the QDSL does not form a miniband at temperatures below ~40 K. If the GS is filled with electrons by doping, photoexcited electrons can drift in the ES miniband without relaxing into the GS. Or, if the GS forms the miniband, the carriers are remarkably separated in the GS miniband. According to these efforts, the electron lifetime would become longer, and the electric field exhibiting the peak ΔEQE approaches the optimal point.

The two-photon absorption reported here is insufficient even at the low temperature at which thermal carrier escape from the IB is suppressed. This is due to the weak VB-to-IB interband and IB-to-CB intersubband absorption strengths. The interband absorption strength is proportional to the QD density. The intersubband absorption strength is limited by the optical selection rule and the electron density of the initial state of the intersubband transition. In order to improve this intersubband transition strength, a photon ratchet IBSC has been proposed, in which the charge carriers in the IB quickly move to the ratchet band where they may have a long lifetime since the ratchet band is optically isolated from the VB^18^. The final state of the first interband excitation is different from the initial state of the second intersubband excitation. Our results indicate that excited carriers in the SL miniband are spatially separated by the internal electric field, so that the electron lifetime in the IB increases with reducing the electron-hole recombination rate. The long-lived electrons in the intermediate states of the SL miniband increase the intersubband absorption strength. This picture is very similar to the concept of the photon ratchet IBSC. Thereby, it is to be desired that the VB-to-IB interband absorption strength becomes stronger. On the other hand, at room temperature, thermal carrier escape from QDs becomes significant and reduces the carrier density in the IB, so that the two-photon absorption becomes weak. The thermal carrier escape can be suppressed by introducing a high potential barrier such as Al_*x*_Ga_1−*x*_As. Recently, we have studied effects of an increased barrier height for InAs/GaAs QDs sandwiched by a high potential barrier of Al_0.3_Ga_0.7_As on the two-step photocarrier generation^15, 16^. We demonstrated remarkable two-step photocarrier generation at room-temperature. QDs in a quantum-well structure enable to increase the VB-to-IB interband absorption strength, and the high potential barrier for electron dramatically reduces the thermal escape. According to these results, the spatial carrier separation and introduction of a high potential barrier are essential to achieve a device operation in realistic conditions.

## Methods

We fabricated a *p-i-n* GaAs based IBSC structure using solid-source molecular beam epitaxy. The As_2_ beam-equivalent pressure was 1.3 × 10^−3^ Pa. Undoped 9- stacked InAs/GaAs QDSL layers were included in the intrinsic layer after growing *n*-GaAs(Si: 5 × 10^17^/cm^3^)/*n* ^+^-GaAs(Si: 1 × 10^18^/cm^3^) layers on *n* ^+^-GaAs(001) substrate at 550 °C. The total thickness of the intrinsic layer of the IBSC structure was 2 μm, which comprised undoped-GaAs (674 nm)/QDSL (38 nm)/undoped-GaAs (1,290 nm). The substrate temperature during the intrinsic layer growth was 480 °C. The nominal thickness of the first QD layer was 2.0 monolayers. The in-plane density of QDs was ~1 × 10^10^/cm^2^, as estimated using atomic force microscopy imaging^12^. We supplied a reduced amount of 1.4 monolayers of InAs for the stacking growth, to prevent a change in the lateral size of the stacked QDs. The thickness of the GaAs spacer layer separating the InAs QD layers was 4 nm, allowing the coupling of electronic states along the stacking direction. When the homogeneous linewidth is larger than the inhomogeneous one along the stacking direction, electronic states can couple, forming a miniband. Here, the GS of the QDSL did not form a miniband at temperatures below ~40 K, because the homogeneous linewidth producing the electronic coupling was smaller than the inhomogeneous distribution of the GS along the stacking direction^12, 14, 28^. Conversely, the homogeneous linewidth of the ES was more than one order of magnitude larger than that of the GS, owing to the weaker electron confinement in the ES^29^. Such a wide homogeneous linewidth of the ES easily allows miniband formation even at low temperatures, as the GS does not form a miniband. This enables photoexcited electrons and holes to transport in the ES miniband in an electric field. Independent energy relaxation of electrons and holes into the GSs located along the stacking direction reduces the recombination rate and extends the electron lifetime^14^, improving the intersubband transition strength. The internal electric field intensity in the QDSLs was expected to be 7 kV/cm, sufficiently low for preventing carrier escape^13^. Finally, a *p* ^+^-GaAs (Be: 1 × 10^19^/cm^3^)/*p*-GaAs (Be: 2 × 10^18^/cm^3^) layer was grown on it at 500 °C. Metal contacts on the top and back surfaces of SCs were Au/Au-Zn and In, respectively.

We performed photocurrent spectroscopy at 9 K. For the interband excitation, we used a halogen lamp dispersed by a monochromator with a focal length of 250 mm. The excitation wavelength was varied in the 600–1,100 nm range, and the excitation density was controlled to be in the 6.36 × 10^13^–8.98 × 10^14^ photons/cm^2^ range (corresponding to 21.1–176 μW/cm^2^). We obtained the EQE spectrum as a measured photocurrent divided by the flux of incident photons. Two-step photocurrent measurements were conducted under two-colour photoexcitation of an additional IR light source with 0.5 eV (2,480 nm) for the second intersubband excitation as well as the interband excitation mentioned above. We used an optical parametric amplifier with the repetition rate of 200 kHz for the IR light source. The excitation power density was 6.5 × 10^18^ photons/cm^2^ (520 mW/cm^2^), which is equivalent to 60 suns. The excitation energy of 0.5 eV was sufficient for causing the IB-to-CB intersubband transition^30^, because the confined potential energy for electrons in the GS of QDSLs was estimated to be 0.26 eV based on the temperature dependence of the PL intensity. The IR light generated by the optical parametric amplifier was chopped at 1.8 kHz, and the modulated photocurrent was detected using a lock-in amplifier synchronised at the chopped frequency. Usually, ΔEQE is defined as a change in the EQE signal amplitude modulated by the IR illumination. In this study, we systematically measured EQE and ΔEQE for different electric fields, controlled by applying a DC bias voltage. We compared the experimental results with theoretical calculations taking into account rate equations describing transitions in the QDSL states.
